# Performance of ChatGPT on questions from the Brazilian College of Radiology annual resident evaluation test

**DOI:** 10.1590/0100-3984.2023.0083-en

**Published:** 2024-03-25

**Authors:** Cleverson Alex Leitão, Gabriel Lucca de Oliveira Salvador, Leda Maria Rabelo, Dante Luiz Escuissato

**Affiliations:** 1 Universidade Federal do Paraná (UFPR), Curitiba, PR, Brazil

**Keywords:** Artificial intelligence, Radiology, Examination questions, Diagnostic imaging, Inteligência artificial, Radiologia, Questões de prova, Diagnóstico por imagem

## Abstract

**Objective:**

To test the performance of ChatGPT on radiology questions formulated by the Colégio Brasileiro de Radiologia (CBR, Brazilian College of Radiology), evaluating its failures and successes.

**Materials and Methods:**

165 questions from the CBR annual resident assessment (2018, 2019, and 2022) were presented to ChatGPT. For statistical analysis, the questions were divided by the type of cognitive skills assessed (lower or higher order), by topic (physics or clinical), by subspecialty, by style (description of a clinical finding or sign, clinical management of a case, application of a concept, calculation/classification of findings, correlations between diseases, or anatomy), and by target academic year (all, second/third year, or third year only).

**Results:**

ChatGPT answered 88 (53.3%) of the questions correctly. It performed significantly better on the questions assessing lower-order cognitive skills than on those assessing higher-order cognitive skills, providing the correct answer on 38 (64.4%) of 59 questions and on only 50 (47.2%) of 106 questions, respectively (*p* = 0.01). The accuracy rate was significantly higher for physics questions than for clinical questions, correct answers being provided for 18 (90.0%) of 20 physics questions and for 70 (48.3%) of 145 clinical questions (*p* = 0.02). There was no significant difference in performance among the subspecialties or among the academic years (*p* > 0.05).

**Conclusion:**

Even without dedicated training in this field, ChatGPT demonstrates reasonable performance, albeit still insufficient for approval, on radiology questions formulated by the CBR.

## Abstract (pt)

**Objetivo:**

Testar o desempenho do ChatGPT em questões de radiologia formuladas pelo Colégio Brasileiro de Radiologia (CBR), avaliando seus erros e acertos.

**Materiais e Métodos:**

165 questões da avaliação anual dos residentes do CBR (2018, 2019 e 2022) foram apresentadas ao ChatGPT. Elas foram divididas, para análise estatística, em questões que avaliavam habilidades cognitivas de ordem superior ou inferior e de acordo com a subespecialidade, o tipo da questão (descrição de um achado clínico ou sinal, manejo clínico de um doente, aplicação de um conceito, cálculo ou classificação dos achados descritos, associação entre doenças ou anatomia) e o ano da residência (R1, R2 ou R3).

**Resultados:**

O ChatGPT acertou 53,3% das questões (88/165). Houve diferença estatística entre o desempenho em questões de ordem cognitiva inferior (64,4%; 38/59) e superior (47,2%; 50/106) (*p* = 0,01). Houve maior índice de acertos em física (90,0%; 18/20) do que em questões clínicas (48,3%; 70/145) (*p* = 0,02). Não houve diferença significativa de desempenho entre subespecialidades ou ano de residência (*p* > 0,05).

**Conclusão:**

Mesmo sem treinamento dedicado a essa área, o ChatGPT apresenta desempenho razoável, mas ainda insuficiente para aprovação, em questões de radiologia formuladas pelo CBR.

## INTRODUCTION

Artificial intelligence (AI) is the general name given to computing methods that simulate the learning pattern of the human brain^(1)^. The rapid advances recently made in this field of knowledge have raised questions about how it will impact diverse professions, including that of medicine, in the future. Among the existing AI models, the Chat Generative Pretrained Transformer (ChatGPT) has gained prominence, not only in the scientific literature^(2-4)^ but also in the popular media^(5)^. It is an AI tool based on the relationships between AI algorithms and human language, a strategy known as natural language processing, and has been publicly available since November 30, 2022^(6)^. Its current model is GPT-3.5, a large language model trained on more than 45 terabytes of textual data. Through neural networks, those data give the tool the capacity to analyze texts and generate texts similar to those written by humans^(7)^. Although it has not been specifically trained for medical use, studies have demonstrated its promising role in medical practice^(8)^ and in academic medical writing^(9)^. As a way of evaluating the knowledge of ChatGPT on medical topics, its performance has been tested on academic examinations that evaluate real students, such as the test for obtaining a medical license in the United States^(10)^, and on questions for obtaining specialist degrees in radiology in Canada and the United States^(7)^, as well as on those for obtaining a degree in family medicine in Taiwan^(11)^, with results that show its performance to be, in general, close to that required for approval.

In the specific context of radiology, AI has been used mainly as an aid in image interpretation, although language models such as ChatGPT have also shown potential as an aid in writing radiological reports^(12)^ and in clinical decision making^(4)^. A better understanding of the performance of AI in the context of problems encountered in daily radiology practice can help us understand how it will influence the future of the profession. With that objective in mind, we sought to evaluate the performance of ChatGPT on questions prepared by the *Colégio Brasileiro de Radiologia* (CBR, Brazilian College of Radiology) for the annual evaluation of residents in radiology and diagnostic imaging, analyzing its answers to determine what its current strengths and weaknesses are.

## MATERIALS AND METHODS

This was a prospective analytical study carried out between May 24 and June 3 of 2023. Because the study did not involve human beings or patient data, approval by an institutional review board was not required.

### Questions for the annual evaluation of radiology residents

A total of 165 questions were selected from the annual evaluation tests for residents in radiology and diagnostic imaging applied by the CBR in the years 2018, 2019, and 2022, which are available online for public access on the CBR website^(13)^ and whose use has been authorized by the CBR Committee for Certification and Licensing. All questions were of the multiple-choice type, with only one correct answer and four incorrect answers. Questions with images were excluded, because ChatGPT does not yet have the ability to interpret images. They were divided according to their topic into physics questions (n = 20) and clinical questions (n = 145), the latter representing the main fields of knowledge and subspecialties of radiology: abdominal imaging (n = 20); thoracic imaging (n = 15); breast imaging (n = 15); neuroradiology (n = 15); pediatric radiology (n = 15); musculoskeletal imaging (n = 15); contrast media (n = 15); ultrasound (n = 15); obstetrics and gynecological imaging (n = 10); and miscellaneous, including positron-emission tomography/computed tomography, densitometry, Doppler ultrasound, and radiation safety (n = 10).

Subsequently, the questions were subdivided, according to the principles of Bloom’s taxonomy^(14)^, into questions that assess lower-order cognitive skills (remember an idea, memorize a concept) and questions that assess higher-order cognitive skills (evaluate, analyze, synthesize knowledge obtained). Those that assess higher-order cognitive skills were again divided, by style, into six subcategories: description of a clinical finding or sign; clinical management of a case; application of a concept; calculation or classification of the findings described; correlations between diseases; and anatomy. Each of the authors, working independently, classified all of the questions. In cases of disagreement, the final classification was obtained by consensus.

Finally, the questions were divided into three tiers: those applied to all residents (n = 92); those applied to secondand third-year residents (n = 34); and those applied to third-year residents only (n = 39).

### ChatGPT

The most recent version of ChatGPT available (May 24, 2023; OpenAI) was used. Although this tool was trained with more than 45 terabytes of data in text forma (from web pages, books, and scientific articles), those data were not provided specifically to meet the needs of the radiologist. ChatGPT does not perform internet searches; it answers questions using only its own database.

### Data collection and analysis

The questions and their respective answer choices were presented to ChatGPT sequentially, one by one, exactly as formulated by the CBR, without providing a specific pre-prompt, and its answers were saved in a text file for later analysis by the researchers. For the questions it answered incorrectly, feedback was provided immediately, the error being explained and the correct answer being supplied, in order to analyze the behavior of the model in response to the correction. In addition to the quantitative analysis of the numbers of correct and incorrect answers, the researchers carried out a qualitative group analysis, obtaining a consensus for comments regarding the answers given.

### Statistical analysis

To analyze the accuracy rate, the ratio between the number of correct answers and the total number of questions was calculated for all categories (overall; highand low-order questions; and the question subtypes as described above). The final (overall) ratio was converted to a percentage to represent the accuracy rate.

Comparisons between the question groups (low-order vs. high-order cognitive skills; physical vs. clinical; and one style vs. another style) in terms of the accuracy rate were made by using Fisher’s exact test or the chi-square test, as appropriate. The analysis among subgroups of questions (by topic and target academic year) was performed with analysis of variance. The statistical analysis was performed with Stata software, version 16.0 (Stata Corp LP, College Station, TX, USA), and post-processing was carried out by using the Analyze Data feature of Microsoft Excel 365. Values of *p* < 0.05 were considered statistically significant.

## RESULTS

### Overall result

ChatGPT provided a correct answer on 88 of the 165 questions asked, resulting in a score of 53%, which is well below the 70% defined as a passing score by the CBR. Table 1 shows its performance according to the type and topic of the question.

**Table 1:** ChatGPT performance by question type and topic.

Question characteristic	Questions n	Correct answers n (%)	*P*
Type			
Lower-order cognitive skills	59	38 (64.4)	
Higher-order cognitive skills	106	50 (47.2)	
Description of findings	42	22 (52.4)	0.81^*^
Clinical management	22	12 (54.5)	0.72^*^
Application of a concept	57	38 (66.7)	0.67^*^
Calculation/classification of findings	8	3 (37.5)	0.92^*^
Correlations between diseases	26	11 (42.3)	0.63^*^
Anatomy	10	2 (20.0)	0.58^*^
Topic			
Physical	20	18 (90.0)	0.02^*^
Clinical	145	68 (46.8)	0.41^*^
Abdominal imaging	20	13 (65.0)	0.62^†^
Thoracic imaging	15	9 (60.0)	0.56^†^
Neuroradiology	15	5 (33.3)	0.76^†^
Musculoskeletal imaging	15	8 (53.3)	0.87^†^
Breast imaging	15	7 (46.7)	0.61^†^
Contrast media	15	9 (60.0)	0.94^†^
Ultrasound	15	3 (20.0)	0.78^†^
Pediatric radiology	15	10 (66.7)	0.93^†^
Obstetrics and gynecological imaging	10	2 (20.0)	0.72^†^
Miscellaneous	10	4 (40.0)	0.65^†^
Total	165	88 (53.3)	0.01^*^

* Fisher’s exact test.

† Analysis of variance.

### Performance by question type

**Figure 1:**
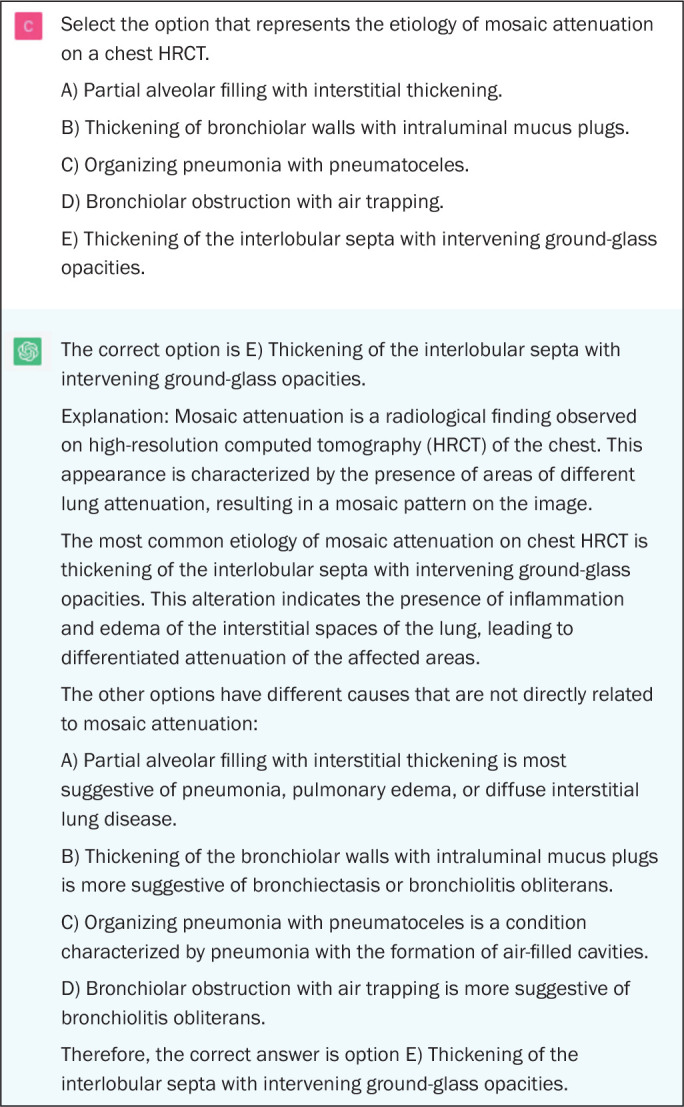
Example of a correct answer provided by ChatGPT on a question assessing a lower-order cognitive skill (a definition, in this case).

**Figure 2:**
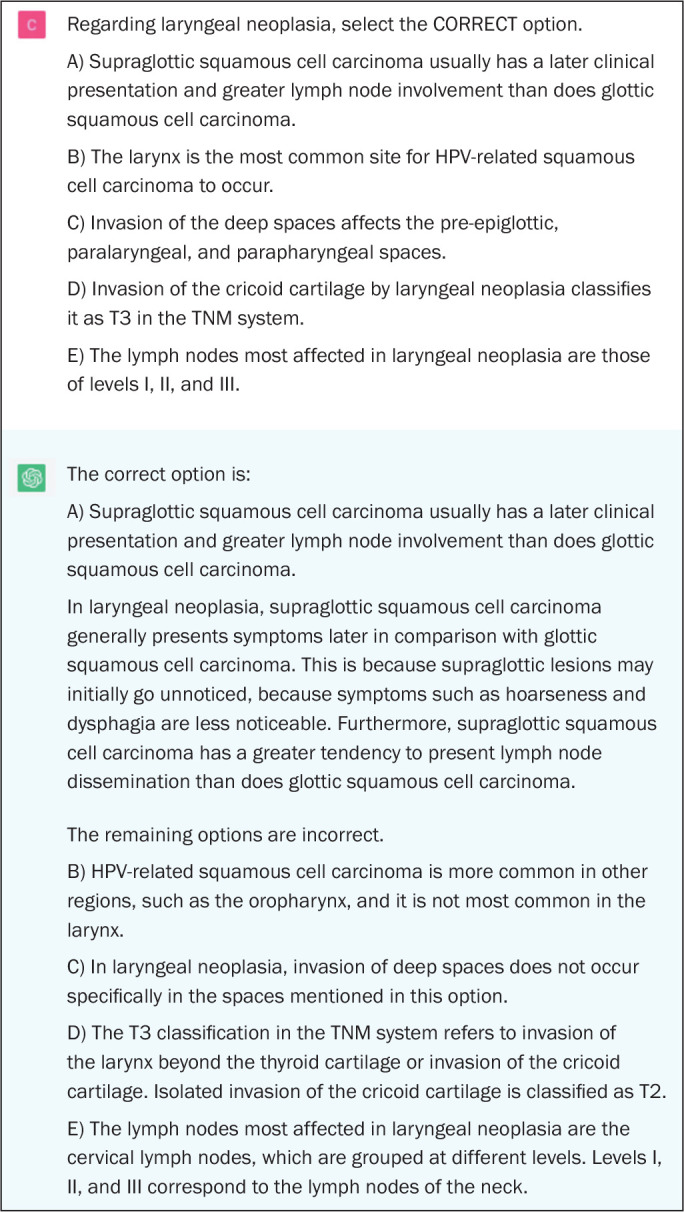
Example of a correct answer provided by ChatGPT on a question assessing a higher-order cognitive skill (the correlation between diseases, in this case).

The performance of ChatGPT was better on questions that assess lower-order cognitive skills, for which it provided the correct answer on 38 (64.4%) of the 59 questions, than on questions that assess higher-order cognitive skills, for which it provided the correct answer on only 50 (47.2%) of the 106 questions, and the difference was statistically significant (*p =* 0.01). Figures 1 and 2 show examples of correct answers on questions that assess lowerand higher-order cognitive skills, respectively.

**Figure 3:**
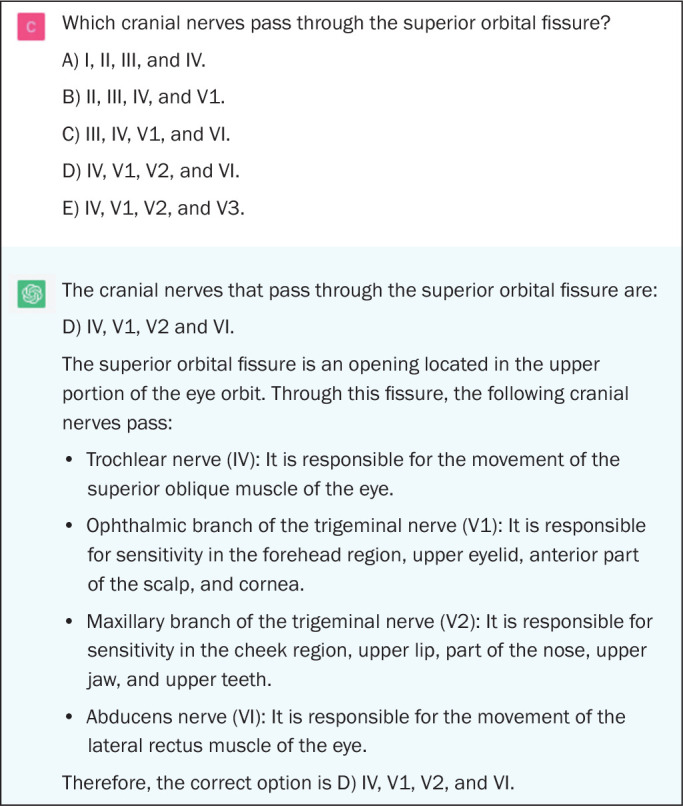
Example of a ChatGPT error on an anatomy question in neuroradiology. The correct answer would be C.

**Figure 4:**
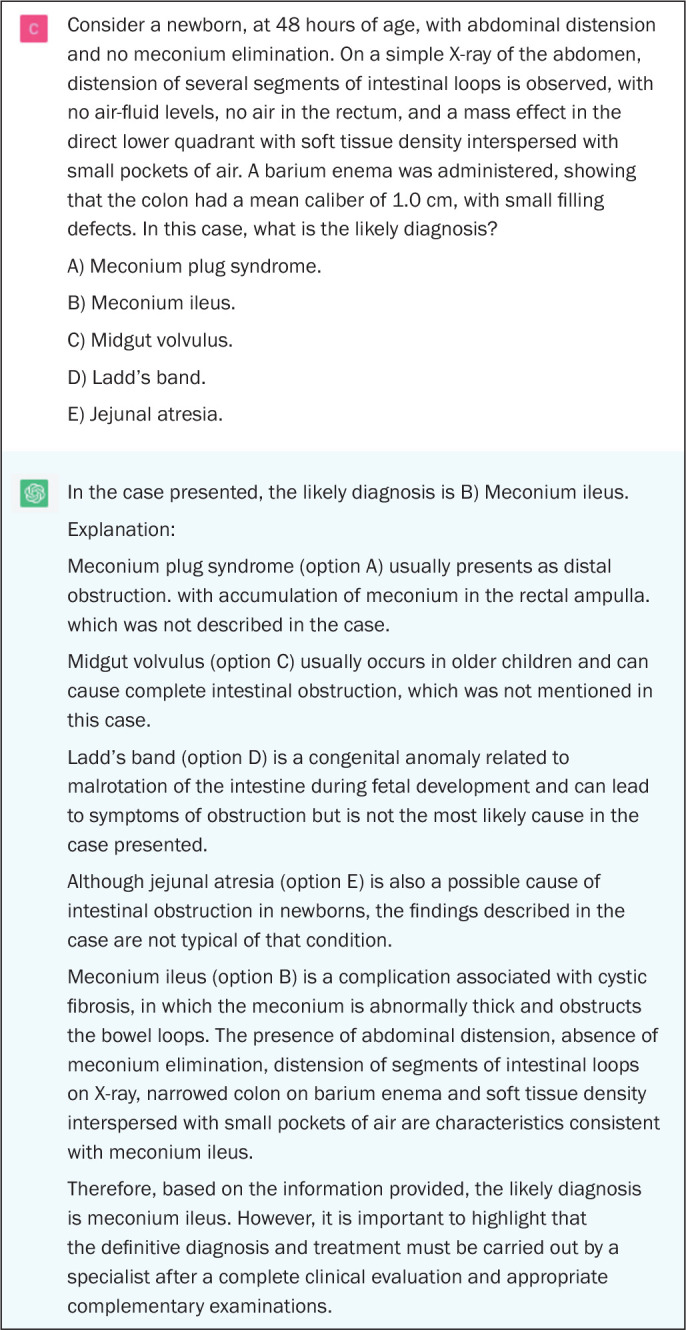
Example of a correct answer provided by ChatGPT on a question in which there is a description of the imaging findings and a diagnosis is requested.

**Figure 5:**
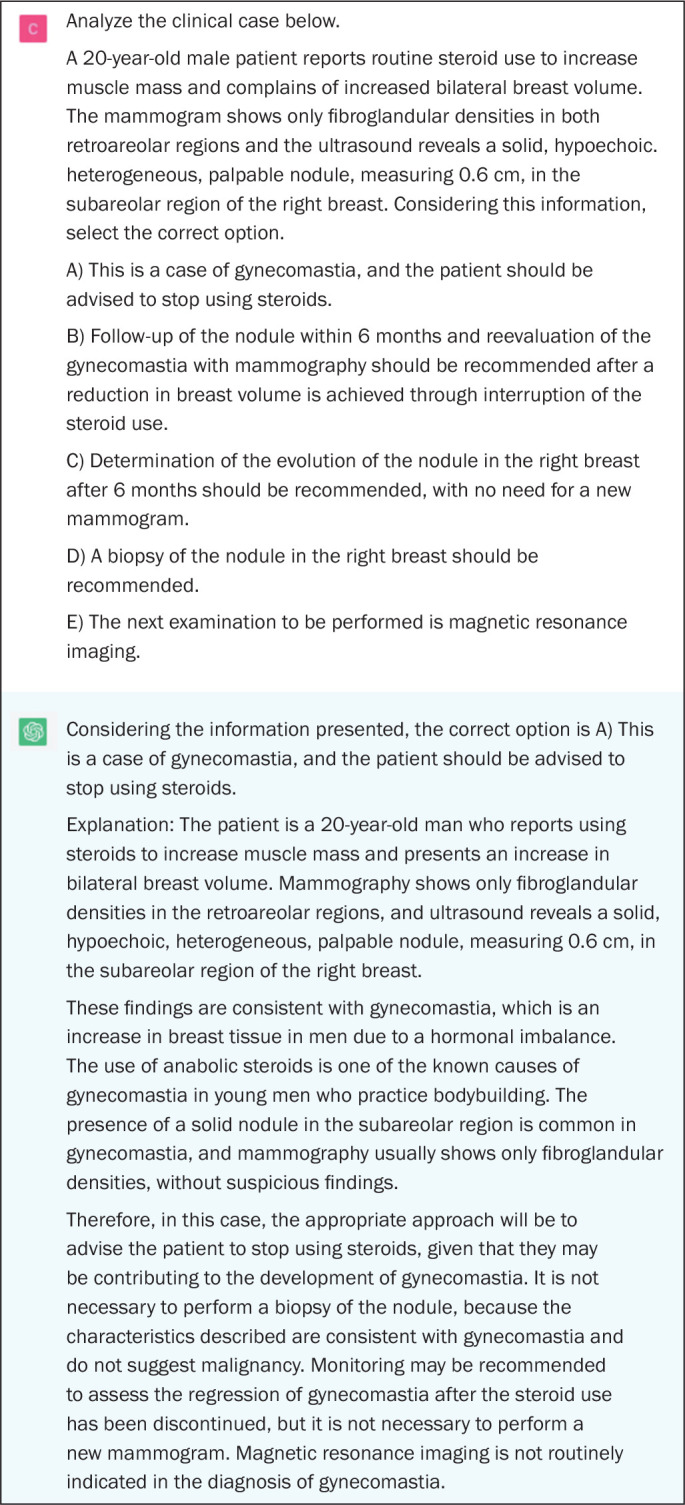
Example of a correct answer provided by ChatGPT on a question in which there is a description of a clinical case with imaging examination and the most appropriate course of action is requested.

Among the questions that assess higher-order cognitive skills, the performance of ChatGPT was poorer on those related to anatomy, calculation/classification, and correlations between diseases, although there was no statistically significant difference in comparison with the questions on which it performed better, which were those related to the description of findings, clinical management, and application of concepts (*p* > 0.05). Figure 3 shows an example of a ChatGPT error on a question regarding anatomy, Figure 4 shows an example of a correct answer on a question regarding the description of findings, and Figure 5 shows an example of a correct answer on a question regarding clinical management.

### Performance by question topic

ChatGPT performed better on physics questions than on clinical questions, and the difference was statistically significant (*p* = 0.02). Among the clinical questions, the accuracy rates were highest for the questions on pediatric radiology, abdominal imaging, and thoracic imaging, although there was no statistically significant difference in comparison with the questions on obstetrics/gynecological imaging and ultrasound, for which the accuracy rates were lowest (*p* > 0.05).

### Performance by target academic year

ChatGPT performed best on the questions applied to all residents, providing a correct answer on 57 (61.9%) of the 92 questions, followed by those applied to secondand third-year residents, for which it provided a correct answer on 17 (50.0%) of the 34 questions and those applied to third-year residents only, for which it provided a correct answer on 14 (36.9%) of the 39 questions. However, there was no statistically significant difference among the categories (*p* > 0.05).

### Qualitative assessment of the answers

The unanimous assessment of the evaluators was that the performance of ChatGPT was satisfactory, especially given that its database was not developed specifically for use in the field of radiology. The high degree of assertiveness that the model exhibited in providing its answers, never using words that would indicate doubt or hesitation (Figures 1 to 5), even in answers that were incorrect (Figure 3), was also noteworthy. Another interesting finding is that, on 107 (64.8%) of the 165 questions, the model not only indicated the correct answer but also analyzed all of the other answer choices, indicating why it judged them to be incorrect (Figures 1, 2, and 4).

## DISCUSSION

To our knowledge, this is the first study of its type to be carried out exclusively with data related to Brazil. Our findings make it evident that the accuracy of ChatGPT on radiology questions is not yet high enough to obtain the score required for approval on the annual CBR evaluation of residents in radiology and diagnostic imaging. The performance of ChatGPT on questions designed for radiology residents in Brazil was worse than that observed on questions designed for their counterparts in Canada and the United States^(7)^-53.3% versus 69.0%-which might be attributable to differences between the two tests in terms of the specific knowledge that each country demands from its future radiologists. New, similar studies carried out in other countries might clarify such differences.

The analysis of the 77 questions that ChatGPT got wrong shows that its errors can basically be attributed to a lack of knowledge of the subject being addressed, as exemplified in Figure 3. No errors in interpretation of the statement, illogical associations, or so-called hallucinations were identified. This result is in line with what is described in the literature, which shows that hallucinations are not as frequent in chatbots because they are designed to answer questions based on rules established during the programming phase and on the information contained in their databases, rather than to generate new information^(15)^, which is usually the source of hallucinations. A similar study recently confirmed that tendency^(16)^, which suggests that chatbots lack familiarity with the specificities and nuances of radiology, that lack of familiarity being the main obstacle to achieving higher accuracy rates.

The fact that ChatGPT performs better on questions that assess lower-order cognitive skills than on those that assess higher-order cognitive skills has been demonstrated in the literature^(7)^ and was corroborated in the present study. This finding shows the ability of AI to recognize and express concepts and definitions while indicating that there are still advances to be made in terms of meeting more complex challenges. It is important that this characteristic of current AI models be known, so that future efforts can be directed toward increasing their performance in both orders of cognitive skills.

Large language models like ChatGPT are trained, from a large database, to recognize language patterns and the relationships between words. Therefore, the superior accuracy rate for physics questions over clinical questions observed in the present study is understandable. Because the ChatGPT database was not created specifically to meet the needs of radiologists, other areas of knowledge that transcend this specialty, such as physics, have the potential to generate a greater number of associations, thus increasing the accuracy rate for the challenges proposed. Such language models, including ChatGPT, could benefit from greater training in this medical specialty in the future. However, until then, it is important that radiologists be aware of this limitation.

Likewise, the absence of a statistically significant difference between radiology subspecialties can be understood as resulting from the limited familiarity that ChatGPT has with the terms and jargon employed in each of those areas. Radiology and each of its subspecialties have their own vernacular that is used in preparing reports, making classifications, and describing diagnoses. As long as the large language model database is not specifically trained to deal with these terms, the AI can be led to make incorrect associations, which limits its accuracy. For example, the word “density” has an obvious meaning for the radiologist, but it can be recognized by ChatGPT as a different concept from that intended, simply because of the lack of training with the term in the specific context. Training in this specific technical language could improve the accuracy of AI, not only in radiology as a whole but also in its subspecialties.

Another noteworthy finding of the present study is the fact that ChatGPT analyzed all of the alternative answer choices for most of the questions presented. It is not clear what factor motivated the model to carry out such an analysis for some questions and not for others, given that the phenomenon was observed for questions related to all specialties and of all types, regardless of their characteristics. Nevertheless, when the analysis of the alternative answer choices is not done spontaneously, it is possible to ask ChatGPT in a subsequent message to carry out such an evaluation, and those requests were complied with 100% of the time in our study. This is a skill that can become useful for residents who wish to use the questions from previous tests, which are made available by the CBR, as study material. More than simply indicating the correct answer, the model tends to provide a complete study of the statements that make up the question, reviewing the topics covered in it, which indicates a possible role for ChatGPT as an auxiliary study tool, capable of succinctly yet efficiently reviewing topics of interest to radiology residents.

One of the differences between our findings and those of similar studies carried out in other countries is that the proportion of correct answers on questions related to the topic of physics was relatively high in our study. For example, ChatGPT provided the correct answer on 90% of the physics questions in our study, compared with only 40% in a study carried out in the United States^(7)^. Although it cannot be said with certainty, it is possible that the divergence is attributable to differences in the content of the questions (variations between the two countries in terms of the topics that are addressed within the field of physics) or in the process of their formulation (in this study, they were created by a specialized committee of the CBR, which is a national institution, whereas, in the study conducted in the United States case, the questions were created by researchers at a single center). In addition, although it is not yet clear, it is possible that the source language also has some influence on the performance of ChatGPT, given that there is greater availability of literature in English for training the model, which would therefore, theoretically, have less familiarity with questions in Portuguese. Furthermore, the translation performed by the model may not perfectly capture the meaning of some of the natural terms or expressions in Portuguese. As new studies in different languages appear, it is hoped that this topic will be elucidated.

This study has some limitations. Only objective, theoretical questions that did not involve the interpretation of radiological images were used, because ChatGPT does not yet have the capability to interpret images. The fact that that we provided feedback (correction) after each error might have had an influence on the performance of ChatGPT; it is possible that its subsequent answers would have been different if there had been no such feedback. How much this interaction with the model affects the final result is a line of research that has yet to be explored. In addition, the number of questions related to each subspecialty was relatively small, which limits the comparison between these groups. Future studies with a greater number of questions could enrich this discussion.

## CONCLUSION

In summary, this study shows that, even without dedicated training in this area, ChatGPT presents reasonable performance, albeit still insufficient for approval, on radiology questions formulated by the CBR. It is expected that specific training in radiology for AI models such as ChatGPT will make their performance in matters of this specialty progressively better, and the radiology community must remain attentive to this evolution in order to take advantage of its potential.
